# Hemopexin and haptoglobin: allies against heme toxicity from hemoglobin not contenders

**DOI:** 10.3389/fphys.2015.00187

**Published:** 2015-06-30

**Authors:** Ann Smith, Russell J. McCulloh

**Affiliations:** ^1^School of Biological Sciences, University of Missouri-Kansas CityKansas City, MO, USA; ^2^Pediatric and Adult Infectious Diseases, Children's Mercy-Kansas CityKansas City, MO, USA; ^3^School of Medicine, University of Missouri-Kansas CityKansas City, MO, USA

**Keywords:** hemopexin, heme, haptoglobin, iron, plasma protein therapeutics, hemolytic index, hemolysis, erythrophagocytosis

## Abstract

The goal here is to describe our current understanding of heme metabolism and the deleterious effects of “free” heme on immunological processes, endothelial function, systemic inflammation, and various end-organ tissues (e.g., kidney, lung, liver, etc.), with particular attention paid to the role of hemopexin (HPX). Because heme toxicity is the impetus for much of the pathology in sepsis, sickle cell disease (SCD), and other hemolytic conditions, the biological importance and clinical relevance of HPX, the predominant heme binding protein, is reinforced. A perspective on the function of HPX and haptoglobin (Hp) is presented, updating how these two proteins and their respective receptors act simultaneously to protect the body in clinical conditions that entail hemolysis and/or systemic intravascular (IVH) inflammation. Evidence from longitudinal studies in patients supports that HPX plays a Hp-independent role in genetic and non-genetic hemolytic diseases without the need for global Hp depletion. Evidence also supports that HPX has an important role in the prognosis of complex illnesses characterized predominantly by the presence of hemolysis, such as SCD, sepsis, hemolytic-uremic syndrome, and conditions involving IVH and extravascular hemolysis (EVH), such as that generated by extracorporeal circulation during cardiopulmonary bypass (CPB) and from blood transfusions. We propose that quantitating the amounts of plasma heme, HPX, Hb-Hp, heme-HPX, and heme-albumin levels in various disease states may aid in the diagnosis and treatment of the above-mentioned conditions, which is crucial to developing targeted plasma protein supplementation (i.e., “replenishment”) therapies for patients with heme toxicity due to HPX depletion.

## Introduction

The low, essentially constant leakage of hemoglobin (Hb) from red blood cells (RBCs), termed trivial hemolysis, is well-tolerated; however, both intravascular (IVH) and extravascular (EVH) hemolysis induce severe inflammation and potential tissue dysfunction. This pathology is due to heme (iron-protoporphyrin IX) released from methemoglobin (metHb, Bunn and Jandl, 1968). Unless bound to proteins, the redox-active heme-iron participates in oxygen radical reactions that covalently modify protein, lipid, carbohydrate and/or nucleotides, which then lead to tissue damage. Cellular heme toxicity, first described in 1991 (Balla et al., 1991b), derives partly from heme alone and also through heme-mediated sensitization of cells to subsequent stimuli including reactive oxygen species from activated human neutrophils, inflammatory cytokines, and NO. Cells rely on “extracellular antioxidants,” i.e., the proteins in biological fluids to prevent such radical chemistry by sequestering the heme and its iron (Halliwell and Gutteridge, 1990). Thus, haptoglobin (Hp) binds Hb, the source of heme that is bound by hemopexin (HPX), and transferrin binds iron. In plasma, HPX targets heme to the liver parenchymal cells for heme catabolism, iron storage, and re-distribution. In barrier tissues, HPX targets heme to brain neurons (Hahl et al., 2013). Normally, HPX recycles after heme delivery whereas the Hp-Hb complex is degraded in macrophages. Documented changes in plasma Hp and free Hb levels provide clues as to the extent of hemolysis; but only HPX and heme levels demonstrate problems with heme clearance.

Our purpose is to provide a new perspective on the roles of the HPX and Hp systems in human diseases in which they protect against Hb-derived heme toxicity and body iron loss. HPX is not an acute phase protein in humans, as is Hp, and because of additional differences between these systems in humans and in mice, we focus on reviewing *in vivo* data from human subjects and rhesus monkey research, and *in vitro* data from cultured human cells. Throughout this review, we have used italic font to emphasize key points and conclusions.

Our goal is to describe current understanding of heme metabolism and the deleterious effects of “free” heme on immunological processes, endothelial function, systemic inflammation, and various end-organ tissues (e.g., kidney, lung, liver, etc.), with particular attention paid to the protective role of HPX. We propose that the model of a linear pathway for the development of heme toxicity with HPX as a backup system for Hp, i.e., acting only when Hp becomes depleted, does not represent many situations *in vivo*. Examples from patients and experimental animals provide evidence that HPX and Hp act simultaneously in various niches of the body when hemolysis occurs, both in the presence or absence of infection and inflammation. HPX protects human endothelial (Balla et al., 1993) and hepatic parenchymal (Larsen et al., 2010) cells from direct heme toxicity and heme-sensitization (Balla et al., 1991b). HPX protects human macrophages by limiting the heme-mediated oxidation of lipoproteins (Camejo et al., 1998; Smith, 2013) generating lipid peroxide- and iron loaded-LDL (Balla et al., 1991a). Heme-mediated LDL oxidation contributes to the pathogenesis of atherosclerosis (Camejo et al., 1998; Jeney et al., 2002), heme being derived from ruptured plaques (Nagy et al., 2010) and LDL from the extracellular intima.

Interest in the protective role of HPX has rapidly increased during the past 5 years because the causative role of heme in the pathophysiology of sepsis, sickle cell disease (SCD), other hemolytic conditions and also in atherosclerosis (Balla et al., 1991a; Nagy et al., 2010) has become accepted (Larsen et al., 2010; Smith, 2011a,b; Ghosh et al., 2013; Schaer et al., 2013; Smith, 2013; Belcher et al., 2014; Schaer et al., 2014; Jung et al., 2015). Insights into how HPX acts in clinical IVH and EVH have been obtained primarily through primate-, rat-, mouse-, and human-based studies. *While HPX's role in protection against heme-mediated inflammation and oxidative damage is established, further studies are needed to confirm the Hp-independent activity of HPX in hemolysis in various clinical disease states in humans*. We have therefore summarized and reviewed both historical and recent observations on the HPX and Hp systems in relevant human conditions and diseases. Finally, we propose future research avenues related to heme metabolism in human health and disease. These include quantitating the amounts of plasma heme, HPX, Hb-Hp, heme-HPX, and heme-albumin levels in various disease states to aid in the diagnosis and treatment of diseases prominently characterized by hemolysis and investigating the use of targeted plasma protein supplementation (i.e., “replenishment”) therapies for patients with heme toxicity due to HPX depletion. Although not addressed here, α-1 microglobulin binds heme and free radicals protecting particularly EVH sites (Akerstrom and Gram, 2014), and when infused minimized pre-eclampsia-like symptoms from Hb in perfused human placenta (May et al., 2011); and treated pre-eclampsia in sheep (Wester-Rosenlof et al., 2014).

## Structure and function of hemopexin and haptoglobin and their role in heme and hemoglobin clearance in intravascular hemolysis

The lysis of 1 ml of blood containing 5 billion RBCs each with ~289 million Hb molecules (Sears, 1999) could release ~ 1.45 × 10^18^ Hb molecules, which is 1000-fold higher than 20 μM plasma Hp molecules (6.82 × 10^15^) in the immediate plasma (550 μl). Given that total body plasma Hp would be ~2.8 × 10^19^ molecules (and HPX on average an additional 1.8 × 10^19^ molecules if at 12.4 μM (Drabkin, 1971), it is clear that Hb and heme loads can be massive. Endothelial cells lack HPX receptors (Smith and Morgan, 1979) and unregulated heme diffusion into and metabolism by these cells, estimated to be ~60 trillion cells in the human body (Aird, 2005), represents a formidable asset and heme “sink” but also a major target for heme toxicity.

The crystal structure of the equimolar heme-HPX complex (Paoli et al., 1999) reveals the unique coordination of heme that contributes to one of the highest affinities known, Kd less than pM (Hrkal et al., 1974). Thus, HPX is the key defense against the deleterious effects of heme on cells, particularly hepatic, immune system and endothelial cells. Heme rapidly dissociates from metHb that is quickly generated from HbO_2_A, even faster from sickle Hb (HbS; Hebbel et al., 1988) and also from Hp-bound Hb; particularly in the presence of NO and ROS including hypochlorous acid (Jeney et al., 2002), generated by the respiratory burst of neutrophils and macrophages at sites of inflammation. Ferro-Hb was relatively innocuous to porcine endothelial cells in serum-free medium, whereas ferri-heme, which readily released from globin to which it is bound less tightly was toxic (Balla et al., 1993). The heme globin interaction is stronger in cyano-Hb and in Hp-Hb preventing heme uptake. HPX prevented the metHb toxicity (Balla et al., 1993). Heme sensitizes cells to inflammatory cytokines, e.g., tumor necrosis factor-α (TNF), which is toxic to human hepatocytes (Larsen et al., 2010), and murine peritoneal macrophages (Fortes et al., 2012); as well as to ROS including H_2_O_2_, which predisposes porcine (Balla et al., 1991b) and human umbilical (Jeney et al., 2002) endothelial cells to die. Two synergistic mechanism by which heme causes necrosis of murine peritoneal macrophages are: TNF expression from TLR4/Myd88 pathway activation and TLR4-**in**dependent generation of ROS (Fortes et al., 2012). Micromolar heme activates human neutrophils triggering the oxidative burst and release of the chemokine interleukin 8 (Graca-Souza et al., 2002), resulting in leukocyte migration into tissues. In mice models of SCD, unless HPX is present or replenished, heme causes endothelial cells to present surface adhesion molecules leading to stasis and vaso-occlusion in part via toll-like receptor-4 (TLR4) activation (Belcher et al., 2014) and pulmonary endothelia to produce neutrophil extracellular traps (Chen et al., 2014). Also, HPX down-regulates lipopolysaccharide (LPS)-induced production of TNF and IL-6 inflammatory cytokines in mouse bone marrow-derived macrophages (Liang et al., 2009).

Within RBCs, heme in Hb is maintained in the reduced state needed for oxygen binding. After lysis, this ferro-O_2_ Hb is bound by Hp, then ferro-Hb-Hp and, finally, ferric-Hb-Hp complexes are generated. For Hb transport, each Hp molecule binds the equivalent of one tetramer of Hb [(αβ)_4_], as two αβ globin dimers each monomer with its own heme bound (Andersen et al., 2012). Hp sequesters Hb [Kd 10^−15^ M (Lim et al., 2001)] limiting Hb oxidation with heme release unless ROS are present In humans, Hp-Hb complexes are endocytosed by the cluster of differentiation receptor 163 (CD163) in mature tissue macrophages present in the spleen, liver, lymph nodes, bone marrow, lung, placenta, peritoneum, thymus, and the Kupffer cells of the liver.

Differences between the affinity of human and mouse CD163 for their respective Hb-Hp complexes and for Hb itself indicate that CD163 endocytosis of Hp-Hb (Kd 19 nM) rather than of Hb is preferred in humans in contrast to mice (Etzerodt et al., 2013). Nevertheless, the affinity of human CD163 for Hb (Kd 195 nM) is sufficiently high that the micromolar levels of Hb documented in hemolytic conditions would result in CD163-Hb binding (Receptor occupancy under equilibrium conditions would be expected to be 50% with a ligand concentration at the Kd value, and more than 90% occupancy at 100 × Kd value). Furthermore, mice do not always make good models of certain human inflammatory conditions that accompany hemolysis including trauma, burns, and endotoxemia that have some degree of hemolysis (Seok et al., 2013).

In IVH, the safe clearance of heme and Hb from the plasma by receptor-mediated endocytosis is rapid: within 2–5 min for heme-HPX principally by liver parenchymal cells (Smith and Morgan, 1981) in part via CD91/LRP1 (Hvidberg et al., 2005); and within 10 min for Hb-Hp complexes by macrophage CD163 (Delanghe and Langlois, 2001). Clearance will be impaired when these two scavenger receptors are saturated with any of their many known ligands; and also when Hp and HPX are depleted. Heme is then reserved on human serum albumin (HSA) from which it continuously dissociates. Non-protein bound heme travels at uncontrolled rates across the plasma membrane of cells via diffusion or possibly via channels or interacts with LDL.

The extracellular anti-oxidant protection of cells by HPX and Hp against heme toxicity from Hb, along with their transport function, are only part of the protective responses against heme toxicity for the body. As first shown in 1992 (Balla et al., 1992), the high activities of two intracellular enzymatic systems are key to cell survival from heme toxicity by maintaining safe levels of redox-active heme and iron. Heme oxygenases (HOs) break down heme but in so doing release its iron as the tetrapyrrole ring opens. This chemically reactive iron is rapidly stored within the ferritin molecule via the ferroxidase activity of the H-subunit. Heme and its iron can both be used for biochemical and regulatory purposes or exported. It is now known that heme and iron export systems (FLVCR and FPN1, respectively) and the acceptor proteins, HPX and transferrin, respectively, are also important. Regardless, survival against heme requires that cell metabolism is competent, not compromised in any way, and thus able to provide cofactors including oxygen, NADPH for HOs and obligatory reductases, and also for H-ferritin. Resistance of porcine endothelial cells to heme toxicity and heme sensitization required chronic, rather than acute, exposure due to the induction of HO1, Ferritin, and particularly H-ferritin (Balla et al., 1992, 1993). Significantly, heme and its iron, respectively, are required for coordinate induction of HO1 and H-ferritin. When HO1 is induced by chemical oxidative stress, cyto-protective ferritin is not induced, at least in murine macrophages (Sheftel et al., 2007). *Notably, cellular metabolism is disturbed in severe illness, particularly sepsis (Langley et al., 2013, 2014; Tsalik et al., 2014), which limits the role of HO and ferritin among other protective mechanisms in mitigating oxidative damage*.

## The clinical relevance of hemoglobin, hemopexin, and haptoglobin levels

In humans, both HPX and Hp are developmentally regulated and hepatic synthesis is considered the predominant source of plasma protein. HPX is also produced in several immune-privileged sites including human brain neurons (Morris et al., 1993), neural retina photoreceptor cells, and ganglions (Chen et al., 1998); whereas Hp is in adipocytes, macrophages, and neutrophils^1^ and is induced by TNF-α in differentiated human adipocytes (Wang et al., 2005). As previously reviewed (Smith, 2011b), human HPX and Hp gene regulation have not been extensively studied. Both promoters contain sites and additional regulatory regions for the inflammatory cytokine, IL-6 (Poli et al., 1989) (but unlike Hp, HPX is not an acute phase reactant in humans). The HPX promoter drives brain and liver expression (Tolosano et al., 1996). Monkey and mouse HPX genes are regulated by heme (see Section Improving Animal Models of Human Disease in which Hemopexin and Haptoglobin Play a Protective Role Below). Human hepatoma HepG2 cells secrete Hp but not HPX. Human Hep3B cells may represent fetal, rather than adult, liver (Oliviero et al., 1987). Hp is induced by IL-6 in this line (Oliviero and Cortese, 1989) and also by IL-1 but is independent of IL-6 in HepG2 cells (Baumann et al., 1990).

HPX is very low in newborns and increases (in mg/100 ml) from average of 60 (range 40–70) at 1–12 years to average of 77 (range 66–100) at 20–40 years but then declines at 50–70 years (range 50–80, Hanstein and Muller-Eberhard, 1968).

**Figure 1:**
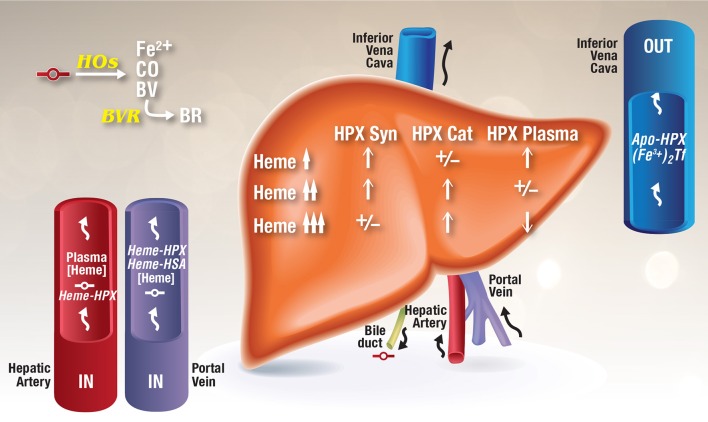
**Model for the development of HPX depletion states**. This model is based on data from rhesus monkeys given heme i.v. (Foidart et al., 1982), HPX metabolism studies in humans (Foidart et al., 1983), known induction of HPX by heme (Smith, 1999; He et al., 2010), normal recycling of HPX after heme delivery (Smith and Morgan, 1978, 1979), and HPX catabolism after i.v. administration of heme several fold higher than the binding capacity of HPX (Sears, 1970; Lane et al., 1972). As heme-HPX forms in plasma, uptake of this complex by receptor-mediated endocytosis into liver parenchymal cells raises heme levels. In HPX deficiency states, heme will traffic unregulated into cells. HOs initially degrade this heme releasing redox active ferrous iron, CO, and biliverdin, which is converted to bilirubin by cytosolic biliverdin reductase. Heme also travels to the nucleus to de-repress Bach 1 target genes including HO-1 and also to induce other genes including the HPX gene. As intracellular levels of heme rise in liver cells in response to increases in plasma heme, changes in HPX synthesis and catabolism are reflected in plasma HPX levels as indicated. Heme is a normal component of bile and as hemolysis progresses, unregulated heme diffusion into cells raises heme levels such that any unmetabolized heme can potentially be exported into the bile (Petryka et al., 1977; McCormack et al., 1982).

The extent of IVH is clinically evaluated from the extent of plasma Hp depletion and increased Hb; plasma heme and HPX are not included. Recent longitudinal studies in patients provide insight into diverse conditions where *a high hemolytic index rate, based principally on plasma Hb, was due to the inability of Hp/CD163 to function normally and clear Hp-Hb*. Thus, in the presence of a high human immunodeficiency virus (HIV) load (Delanghe et al., 2010) HPX was depleted in a patient who presented with symptoms of severe sepsis or hemo-phagocytic syndrome. Blood, plasma, and urine were monitored periodically over 70 days to assess liver, kidney, and muscle function (Delanghe et al., 2010). Free Hb levels were 1.68 g/l (normal <0.11 g/L); Hp levels were 3.37 g/L (reference values 0.3–2.0 g/L), and HPX was essentially undetectable (reference values 0.4–1.5 g/L). After admission, indicators of heme catabolism (i.e., total and direct bilirubin levels) were normal, but blood monocytes expressed slightly lower than normal CD163 levels. Plasma lactate dehydrogenase (LDH), an indicator of tissue damage, was elevated ~10-fold above normal. C-reactive protein was increased and serum creatinine was initially above normal. In the urine, Hb but not heme was detectable. High levels of Hb-Hp complexes were detected in the plasma by starch gel electrophoresis followed by staining for heme (from its peroxidase activity). The circulating Hb-Hp levels declined as the viral load decreased in response to anti-viral therapy, as did C-reactive protein and creatinine, indicating less oxidative stress. The HIV particles likely competed for CD163, limiting Hp-Hb clearance. This patient presented with the Hp2-2 phenotype, shown to confer the worst prognosis of recovery from uncontrolled HIV (Delanghe et al., 1998; Delanghe and Langlois, 2002). The initial abnormally high Hp levels were considered to be due to hepatic induction in response to inflammatory mediators and since infection and inflammation affect monocyte/macrophage function. After an initial decline over the first 6 days they increased to within the normal range at day 16 and nearly doubled by day 70. HPX levels were very low initially (0.1 mg/ml), undetectable at day 6 but increased at day 16 to within the normal range (0.8 mg/ml) and slightly more by day 70 (1.0 mg/ml, Figure 1). Liver and kidney function improved as the viral load declined. These and other data (see Section The Clearance of Hemoglobin by Hemopexin and Haptoglobin during Extravascular Hemolysis, below), demonstrate that key responses in the metabolism and regulation of HPX and Hp are needed to manage a heme load from Hb. *In this patient, HPX and Hp responded individually between day 6 and 16, thus providing an interesting time frame for future studies on plasma protein therapeutics*.

Significantly, heme and iron and their binding proteins HPX and transferrin link nutritional immunity with the innate immune system. Also, CD163 links hemolysis and Hb clearance with the immune response because it acts as a pattern recognition receptor, which binds bacterial surface proteins (Fabriek et al., 2009), and an innate immune sensor for bacterial or local inflammation. Crosslinking of CD163 with antibodies or interaction with bacteria mediates release of pro-inflammatory cytokines (van den Heuvel et al., 1999; Polfliet et al., 2006; Fabriek et al., 2009). Whereas, binding of Hb-Hp1 to CD163 directs the secretion of anti-inflammatory IL10 (Philippidis et al., 2004), although Hb-Hp-2 does not. CD163 induces a cascade of intracellular signals (Ritter et al., 2001) that involves tyrosine kinase-dependent calcium mobilization, inositol triphosphate production, and secretion of inflammatory IL6 and colony stimulating factor 1. Thus, CD163 functions, in an Hp-type dependent manner, to both activate and suppress the innate immune response.

Hb clearance from the plasma by Hp will depend principally upon the number of viable, functioning CD163^+^ monocytes/macrophages in the body. Hb removal was impaired in cells exposed to a drug conjugate of a monoclonal antibody drug compound, gemtuzumab oozogamicin, a biological drug used to treat acute myelogenous leukemia, which also lowers the number of CD163^+^ cells (Maniecki et al., 2008). Perhaps consequently, this compound also increased vaso-occlusive disease in the absence of bone marrow transplantation (Giles et al., 2001). Gemtuzumab has now been withdrawn.

A high hemolysis index in the absence of hemolytic events occurred in response to the metabolism and heme clearance of the second generation Hb-based oxygen carrier blood substitute, pyridoxalated Hb polyoxethylene conjugate (PHP; Drieghe et al., 2013). Hb-based blood substitutes, under development for 40 years^2^, are required for highway accident victims, battlefield casualties, transfusions when an immunological match is not possible or to replace transfused blood refused for religious reasons. Biochemically, they are needed to increase oxygen levels to tissues in the shift from hypoxia to more normoxic conditions in the capillary beds for endothelial cells and the underlying smooth muscle cells to restore their normal metabolism. This process is affected by blood flow rates as well as Hb levels and O_2_ unloading from Hb. Patients respond to decreased O_2_ carrying capacity of blood by increasing cardiac output and ventilation with pulmonary vasodilation, and at the cellular level there is a shift to anaerobic glycolysis. ATP released from RBCs binds to ATP receptors on endothelial cells and induces nitric oxide synthase. NO binding by Hb could cause vasoconstriction with a reduction in blood flow.

In an attempt to minimize the toxic effects of blood substitutes, Hb molecules have been designed to: survive for a long time in the circulation; bind O_2_; have a slow rate of heme autoxidation and scavenge NO minimally. Nevertheless, many blood substitutes are modified Hb molecules with low or no affinity for Hp compared with Hb. Hb or heme released from them into the circulation or interstitial space is potentially toxic. PHP was designed to treat shock associated with the systemic inflammatory response syndrome (SIRS), which is an NO-induced shock with severe hypotension and vasoplegia disrupting major organ function leading to death. PHP has many anti-oxidant properties due to a covalent linkage to catalase and can scavenge NO, and it does not readily oxidize either to metHb derivatives that do not bind O_2_ or to ferryl Hb. It was anticipated to provide treatment in ischemia- reperfusion injury and hemorrhagic shock, where ROS such as superoxide and H_2_O_2_ and NO are known to be involved. Three patients treated with PHP (0.25 ml/kg/h for max of 150 h 3.5 days 7 and 8 days peaks hemolysis index 350U, 650, and 900, respectively) were followed daily for 2 weeks. Plasma Hp levels remained normal but HPX was essentially undetectable by immune-nephelometry. Free Hb (measured as PHP Hb with a higher molecular mass than Hb upon starch gel electrophoresis) was detected in the patients undergoing PHP infusions. The patient's Hp could bind Hb because Hb-Hp complexes formed after Hb addition to plasma samples collected several days after the infusion occurred. Throughout this period, total bilirubin, and heme-binding serum α1-microglobulin levels were normal. Ongoing heme catabolism was apparent with total bilirubin levels increased for several days after PHP infusion. One patient had very low HPX levels initially that eventually rose to low normal levels as their elevated serum creatinine decreased slightly over the second week; and hepatic function improved too as the initially abnormally high aspartate transaminase and alanine amino transferases tended to decline in the second week. However, lactate dehydrogenase (LDH) levels were extremely high initially, increasing over days 3–10 then declining by days 12 and 14. In another patient whose HPX hardly responded, the LDH levels remained abnormally high. *This group of patients provide an example of heme clearance by HPX with an increased hemolysis index value but with normal Hp*.

HPX will be important in clearing myoglobin (Mb)-heme from injured muscle in crush injuries. Mb is expressed in cardiac myocyte and smooth muscle cells. Mb levels, normally 30–90 ng/ml, increase 5- to 10-fold (~500 ng/ml) after myocardial infarcts (Almog et al., 1987; Sallach et al., 2004) and in neuromuscular disease (Foidart et al., 1983). In chronic neuromuscular conditions, heme from Mb affects hepatic HPX metabolism, as does heme and Hb, in a dose-dependent manner (see Section Heme Toxicity and Hemopexin Deficiency States and Refs. Foidart et al., 1982, 1983). In fulminant rhabdomyolosis, HPX is depleted and Mb levels are high, while Hp and Hb are normal implicating HPX in the clearance of circulating Mb (Adornato et al., 1978). Human Hp binds Hb in preference to Mb (Sakata et al., 1986). In Duchenne's muscular dystrophy and polymyositis where there is abnormal Mb metabolism HPX levels were high.

*These patient studies justify adding heme and HPX levels to the hemolytic index (see Figure 1)*; and low HPX levels with elevated LDH may help to distinguish impaired heme plasma clearance from ongoing hemolysis (Drieghe et al., 2013).

## The clearance of hemoglobin by hemopexin and haptoglobin during extravascular hemolysis

Mature RBCs will travel ~300 miles through the systemic vasculature during their 120 day lifespan enduring deformation at every twist, bifurcation, and turn, as well as exposure to changes in osmotic pressure when passing through the kidney and lung vasculature. This journey is equivalent in length to 11.5 marathon runs, and the RBCs may traverse the heart and its valves ~1,70,000 times. Extra-vascular hemolysis (EVH) is the phagocytosis of senescent, damaged or otherwise abnormal RBCs by macrophages in the spleen, liver, bone marrow, and reticulo-endothelial system. Random hemolysis—or the age-**in**dependent destruction of RBCs -occurs at a basal rate of 0.05–0.5% daily. Higher rates of random hemolysis can result in shorter RBC lifespan as does senescence. The chance of a RBC surviving 100 days is only 61% but only 1% survive 100 days when random hemolysis increases to 5%^3^. A compensatory increase in erythropoietin prevents anemia by increasing RBC production and Hb levels.

Most pathological hemolysis is EVH with the spleen playing a major role. EVH is stimulated after one or more blood transfusions in order to clear any RBCs that have deteriorated upon storage. In this case, EVH and IVH likely occur simultaneously, and, depending upon the quality of the stored blood and the number of transfusions, the hemolysis can expose cells and tissues to a significant amount of Hb, heme, and iron. Transfusions can be extensive, for example, car accident victims may require 100 pints of blood, and there is the potential for a daily transfusion need during chemotherapy and a lifetime need in SCD^4^.

After engulfment of RBCs by macrophages, the heme from Hb leaves the endosomes and reaches the smooth endoplasmic reticulum (ER) for catabolism by the HOs 1 and 2 (HO1 and HO2) where each heme molecule stoichiometrically releases redox active ferrous iron and carbon monoxide (CO). Heme also moves to the nucleus for gene regulation in part via depression of Bach1-regulated genes, including HO1 and NADP(H):quinone oxido-reductase 1 (NQO1). These molecules protect cells against oxidative stress as levels of redox active heme and iron rise intra-cellularly. The iron from heme catabolism is either used for biochemical purposes including translational regulation, or is stored or exported via ferroportin (FPN1) and delivered to transferrin. Iron is delivered to liver parenchymal cells by transferrin or by macrophage-derived L-ferritin (Moss et al., 1992) from which it may be re-utilized for erythropoiesis depending upon the body's needs. The extent of iron export will be affected by any changes in surface FPN1 levels, including stabilization by the presence of multi-copper oxidases including ceruloplasmin (McCarthy and Kosman, 2014), loss due to proteasome degradation in response to circulating hepcidin (Nemeth et al., 2004) or increase as heme stimulates FPN1 transcription (Marro et al., 2010).

*There is an important role for HPX in EVH after erythrophagocytosis to accept heme exported by FLVCR, a member of the major facilitator family of transporters thus preventing heme accumulation and toxicity* (Smith, 2011a,b). Substrate solutes such as heme will diffuse down a concentration gradient in this type of transporter but heme export requires a plasma protein heme acceptor such as HPX or HSA (Yang et al., 2010). HPX then delivers heme to the liver (Smith and Morgan, 1978, 1979). Erythrophagocytosis induces HO1 and it is established that heme degradation by HOs is protective against heme toxicity in essentially all cell types with ferritin synthesis and effective heme and iron export. Phagocytic activity is “safe” for macrophages when they engulf <1.5 RBCs. Subsequently, 40% of the Hb-iron is released *in vitro* within 24 h. Ingestion of too many RBCs is lethal in part due to iron toxicity (Kondo et al., 1988). However, *in vitro* experiments with macrophage are not carried out in the presence of *in vivo* levels of HPX, HSA or transferrin.

Evidence supports that heme toxicity in macrophages after erythro-phagocytosis develops either when plasma HPX levels are low, presumably with concomitant IVH, or if HO1 activity is compromised. Both scenarios would contribute to raising intracellular heme levels in macrophages (and also in heme-exposed endothelial cells). The catalytic capacity of HOs may become overwhelmed by the amount of intracellular heme or when there are insufficient substrates and coenzymes for catalytic activity (O_2_ and NADPH for the obligatory HO reductase, cytochrome P-450 reductase). Lowering elevated intracellular heme via FLVCR-mediated export to HPX is another solution. *However, this route would be compromised when circulating HPX is low or absent; and exacerbated when the hydrophobic drug binding site in HSA* (Ghuman et al., 2005) *that accommodates heme is occupied by medications*. In these circumstances, α_1_-microglobulin may provide additional sites for heme.

In humans, a *relationship between HO1 activity and HPX metabolism* was first apparent in HO1-deficiency with undetectable HPX levels in a child that succumbed by 6 years (Yachie et al., 1999), as previously reviewed (Smith, 2011b, 2013); and which is recapitulated in part, in HO1^−/−^ mice that also have low HPX (Kovtunovych et al., 2010). In both situations, the key role for macrophage HO1 activity for heme catabolism and subsequent safe iron distribution in the body with minimal inflammation is apparent. Also, extensive LDL oxidation was detected in this patient perhaps from oxidation of cell free Hb (Jeney et al., 2002) and exacerbated by the HPX deficiency. Both HO1-deficient mice and humans are anemic and in the limited number of humans with HO1 deficiency hyposplenia was found (Yachie et al., 1999), although mice develop splenomegaly from progressive changes in the spleen as macrophages become depleted and heme toxicity develops. As murine HO1^−/−^ macrophages undergo erythrophagocytosis, they burst releasing their contents, including presumably heme, into the spleen causing fibrosis in the areas of red pulp. IVH occurs as senescent RBCs release Hb and heme. The few remaining CD163^+^ macrophages in the spleen cannot effectively clear the Hp-Hb complexes as they do normally. The hepatic parenchymal cells and kidney proximal tubules are thought to become new iron storage sites instead of liver and spleen macrophages. A HPX deficiency state develops in 60% of 8 months old HO1^−/−^ mice, while both Hp and HPX mRNA are increased in the liver. HPX mRNA increases in the liver lysates of HO1^−/−^ mice even by 7 weeks of age and HPX mRNA was also detected at extra-hepatic sites, e.g., in the spleen and kidney, likely due to induction by heme (Smith, 1999) or perhaps the inflammatory cytokine, IL-6 (Tolosano et al., 1996). Although, low plasma HPX and high hepatic HPX mRNA may appear discordant (Vinchi et al., 2008; Kovtunovych et al., 2010), the HO1^−/−^ mice likely have high hepatic heme levels; in which case there is no anomaly because high levels of hepatic heme increase HPX catabolism without compensatory HPX synthesis by the liver as shown in humans with hemolytic anemia and in rhesus monkeys (see Section Heme Toxicity and Hemopexin Deficiency States). Bone marrow transplantation successfully replenished normal progenitors of macrophages restoring Kupffer cell activity to the HO1^−/−^ mouse liver (Kovtunovych et al., 2014) and demonstrating a potential therapeutic strategy.

Successive blood transfusions expose endothelial cells to large amounts of Hb and heme with IVH occurring simultaneously with EVH, especially in the initial stages of each transfusion. CD163-mediated endocytosis of Hp-Hb in macrophages requires ATP. How erythro-phagocytosis affects the overall metabolism of macrophages and how long the cells need to recover the membrane components including CD163 at their cell surface is not known in detail. Presumably, significant amounts of energy are utilized and components of the surface membranes such as lipids and proteins including CD163 may be catabolized or re-distributed in the cell. Thus, erythrophagocytosis may decrease CD163 surface expression with deleterious consequences for the endocytosis and uptake of Hp-Hb or possibly other CD163 ligands including Hb in various hemolytic conditions. Under these circumstances, high levels of metHb are expected and protection against heme toxicity by HPX will become significant until there is sufficient replenishment of CD163^+^ mature macrophages. That this is the case in the clinic is suggested by data obtained during cardiopulmonary bypass (CPB) that entails extracorporeal blood circulation, including priming of the circuit with stored blood and potentially also blood transfusions. Patients with compromised liver function, especially liver cirrhosis and a history of alcohol abuse will not readily synthesize HPX (Vladutiu and Kim, 1981), transferrin and Hp—all extracellular anti-oxidants against heme and iron.

Pediatric patients are especially at risk during CPB due to their lower endogenous HPX levels and need to avoid iron loading, which can lead to problems in neurodevelopment later in life, including decreased IQ, motor deficiencies, impaired language skills, and a higher incidence of behavioral and attention problems (Lull et al., 2008). In neonates especially, surgery-related pathology can occur even with great precautions and improved technology. HPX levels were analyzed by immuno-blot in plasma samples taken just before CPB, 5 min into CPB, at the end of CPB and then 1 and 24 h post-CPB. In 5/13 proteins that changed, HPX and the serine protease inter-α-inhibitor 4 decreased; furthermore copper and Hb levels increased while iron decreased. Although on a non-linear timescale, HPX levels dropped 60% at initiation of CPB and remained low until the end of CPB, then increased to about 75% of the initial value. Hb levels increased from 20 to 60 mg/dL during CPB, and had returned to normal by 24 h post CPB. No changes in Hp were reported in this study. *This rapid, dramatic decrease in HPX levels suggests that the primed blood had generated significant levels of heme to deplete circulating HPX levels and that the heme-HPX complex left the circulation via the liver*.

## Studies on patients with hemolytic diseases reveal complex relationships between hemopexin and haptoglobin levels

Heme sequestration by HPX, its extracellular anti-oxidant function, directly protects cells against heme toxicity; furthermore, heme transported to the liver affects HPX metabolism (see Section Improving Animal Models of Human Disease in which Hemopexin and Haptoglobin Play a Protective Role). *Thus, pinpointing when plasma heme levels rise and the response of HPX will aid in the diagnosis, development of targeted plasma therapeutics, and patient treatment*. Plasma HPX levels were first proposed in 1975 to reveal the severity of hemolysis (Muller-Eberhard and Liem, 1975) when grafted heart valves malfunction. HPX infusion therapy for patients with myocardial infarcts was first proposed in 1993 (Muller-Eberhard and Fraig, 1993). It was noted that this would be simpler technically than measuring CO exhalation, the number of deformed RBCs (schistocytes), serum LDH and urine hemosiderin excretion or the half-life of radioactive RBCs as indications of the extent of hemolysis. Also, plasma Hb levels are compromised by hemolysis during blood collection and storage unless special care is taken.

The clinical units for plasma proteins are in mg/100 ml; whereas, molar units and stoichiometry reveal ligand clearance by transport proteins. One molecule of HPX binds one molecule of heme (Drabkin, 1971); based on an average adult HPX plasma level of 770 μg/ml, ~6.3 μg heme would be bound per ml plasma and higher heme levels would deplete HPX unless recycling takes place or rapid compensatory synthesis takes place (Muller-Eberhard et al., 1968). In molar terms and based on heme binding by HPX in mammalian sera, the HPX levels are ~12.44 μM (Drabkin, 1971) = 1.8 × 10^19^ molecules in 2.23 L av. plasma volume based on 55% of 4.7 L blood volume^5^. Normal plasma heme rarely exceed 5 mg/100 ml plasma (~78 μM) using a modification of the benzidine method. At >10 mg heme/100 ml, methemalbumin accounted for ~65% of the plasma heme and HPX was often decreased in such plasma. Patient samples often had >20 mg heme/100 ml and 325 mg/100 ml has been measured in malaria (Sears, 1968). It is generally accepted that plasma Hp is depleted when plasma Hb is >50–200 mg/100 ml. Hb then passes through the kidney glomeruli and some is retained by the proximal tubular cells where the heme-iron is converted to hemosiderin (detected as urinary iron). Tubular cells are later shed into the urine producing hemosiderinuria and unabsorbed Hb is excreted in the urine (hemoglobinuria). *Notably, plasma schistocyte count and urinary hemosiderin, a measure of excess RBC destruction, correlate inversely with HPX levels* (Eyster et al., 1972).

In the early 1960's and early 1970's, the groundwork for our understanding of the changes in plasma HPX levels in response to increased plasma heme during the course of hemolytic events and human inherited hemolytic diseases was laid by Muller-Eberhard's group with additional longitudinal patient studies by Sears. *Although the patient number is relatively small in these studies, key facts about HPX function and metabolism are apparent*. Both groups provided evidence for a complex, non-linear but significant, relationship between plasma HPX and heme levels (but not Hb); whereas Hp levels were related to Hb levels not heme (Muller-Eberhard et al., 1968; Sears, 1968); see also Section Heme Toxicity and Hemopexin Deficiency States). HPX catabolism did not require Hp depletion; thus, Hp and HPX often simultaneously protect against the development of heme toxicity from Hb. As previously reviewed (Smith, 2011b), comparison of the extent of exogenous heme clearance with HPX levels supported that *HPX recycles after heme delivery to the liver (Muller-Eberhard et al., 1968; Drabkin, 1971), which was subsequently shown directly using radioactive heme*-^125^*I-HPX in rats (Smith and Morgan, 1978, 1979) and confirmed in human hepatoma cells (Smith and Hunt, 1990). These findings remain particularly relevant for and important in the development of plasma protein therapeutics with HPX and/or Hp*.

*In many inherited hemolytic diseases, episodes of heme toxicity occur in a background of chronic oxidative stress and inflammation, as in mice models of SCD (see Section Key Roles for Heme Toxicity in the Pathogenesis of Sickle Cell Disease and Protection by HPX)*. *Sepsis can also entail prominent episodes of hemolysis that are ameliorated by HPX supplementation (see Section Key Roles for Heme Toxicity in the Pathogenesis of Sepsis and Protection by Hemopexin)*. Assessment of the association of HPX levels with the presence of specific diseases, response to therapies, and prognostication of clinical outcomes requires longitudinal studies. These involve repeated monitoring of risk factors and/or outcomes, often on a daily basis. Ideally, the controls will possess similar baseline characteristics as cases, with the primary differences being only the exposure of interest. Both HPX and Hp levels can change quickly in response to a hemolytic episode that “resolves” generally within 1–2 weeks. To a certain degree, the early research on HPX and Hp provides a unique window on the unfolding events affecting the metabolism of these proteins during and after hemolysis—and in the presence and absence of infection. The circulating plasma levels of Hp, HPX, and HSA vary quite widely within the normal population, but are stable for a healthy individual. Cohort studies that use data from much larger groups of patients, which share common characteristics and exposures, are the backbone of current proteomics and outcomes research. Large group cohort studies can be carried out prospectively or retrospectively with differing exposures to a suspected factor or treatment; and then observed and tested generally. Given the relatively rapid changes in HPX and Hp levels, frequent sampling may be necessary to identify correlations between changes in HPX and Hp metabolism and a patient's clinical status.

Decreased plasma HPX levels in hereditary hemolytic diseases was first reported in a study of 38 children in 1963 (Muller-Eberhard and Cleve, 1963). Haptoglobinemia, defined by a lack of detectable Hb binding in plasma, was found in spherocytic anemia with HPX deficiency in 50% of the children before splenectomy to reduce hemolysis; after which Hp was restored in 50% of the children and HPX in all of them. Hp was undetectable in 50% thalassemia major subjects but increased in 100% after splenectomy, whereas HPX only increased in one patient. HPX levels in thalassemia minor were apparently normal, which may reflect the lower levels of heme circulating in that condition. In children with SCD, Hp was low in all cases and HPX was found to be decreased in all but one subject. A later report showed that both HPX and Hp are depleted in sickle cell anemia, sickle cell-HbC disease or thalassemia major (Muller-Eberhard et al., 1968). HPX and Hp were depleted simultaneously in two patients with severe hemolytic reactions to blood transfusions, one linked to anti-E antibodies. There are even cases where HPX levels are normal although Hp has disappeared from the plasma (see also Section Structure and Function of Hemopexin and Haptoglobin and their Role in Heme and Hemoglobin Clearance in Intravascular Hemolysis).

*Some early longitudinal studies revealed that HPX levels were restored to normal far more quickly than Hp levels after a hemolytic episode from a variety of causes (Sears, 1968)*. After each acute episode, heme levels generally spiked (~325 mg heme/100 ml for example in malaria) within 24 h decreasing to normal after 6 days and HPX levels start to increase within 24–48 h. However, Hp remained low requiring 14 days to return to normal plasma levels. This was attributed to a slow ongoing destruction of RBCs and/or the absence of one or more sufficient stimuli for *de novo* Hp synthesis. As described in Section The Clinical Relevance of Hemoglobin, Hemopexin, and Haptoglobin Levels, *macrophage clearance of RBCs is vital for heme and iron homeostasis as it minimizes oxidative stress and inflammation from heme toxicity (Kovtunovych et al., 2010). We propose that severe acute or chronic HO1 impairment links decreased macrophage function in presence of EVH and/or IVH eventually depleting HPX*. However, in relapse from falciparum malaria and in renal insufficiency associated with an immediate hemolytic reaction to a blood transfusion (Sears, 1968), HPX and Hp were depleted and both rapidly restored to normal essentially immediately after plasma heme levels declined.

Without obvious changes in HPX levels, Hp became depleted following hemolysis in response to primaquine in erythrocytic glucose 6-phosphate dehydrogenase deficiency and in relapsed pernicious anemia (Sears, 1968). In the latter case, 10 days after vitamin B12 therapy, Hp levels began to increase. Intriguingly, when the patient developed an infection (pharyngitis, fever and strep throat) there was a rapid and marked two-fold increase in Hp levels within 2–4 days *without significant changes in HPX plasma levels, demonstrating that it is not an acute phase protein in humans (Kushner et al., 1972). Such changes would likely not be apparent in patient proteomic studies where large numbers of control and disease groups are compared*.

A HPX deficiency with normal Hp levels (76 mg/100 ml) was seen in hemorrhagic pancreatitis, where plasma heme was high at 47 mg/100 ml (Sears, 1968). Acute hemorrhagic pancreatitis is an acute inflammation of the pancreas frequently accompanied by hemorrhages into the gland itself and into the parenchyma and surrounding tissue. This condition is often fatal due to the development of shock and multi-organ dysfunction/failure, particularly the lungs and liver, as well as the development of disseminated IVH coagulation. The causes generally include bile duct obstruction (in 50% of all cases), and toxicity from glucocorticoids, acetaminophen, and thiazide diuretics (Frey, 1979).

Normal or only slightly decreased HPX levels with low Hp were detected in paroxysmal nocturnal hemoglobinuria and in auto-immune hemolytic anemia (AHA, Muller-Eberhard et al., 1968). In AHA patients, Hp was below the normal levels of 140 μg/ml, reticulocyte counts were high (23–70%; normal levels are 0.5–1.5%) and antibodies to RBCs were present as shown by positive direct and indirect Coombs test reaction. The reticulocyte count indicates how fast RBC precursors, reticulocytes, are made in the bone marrow and released into the blood stream. This is a normal response to blood loss or premature destruction of RBCs as in hemolytic anemias, which would also be reflected by low blood Hb levels. Kidney disease with increased erythropoietin levels will also increase reticulocyte count.

Normal HPX levels with low Hp was also detected in an adult patient with hereditary spherocytosis, which is the most common form of inherited anemia in individuals of Northern European descent. Mild or severe anemia is due to genetic defects in proteins including ankyrin (ANK1 gene) needed for normal flexibility of red blood cell membrane. These misshapen cells are recognized and taken up by the spleen and destroyed, often necessitating multiple blood transfusions. HPX levels were found to be normal (400 μg/ml) and Hp was low but rose ~5-fold to 600 μg/ml after splenectomy (Muller-Eberhard et al., 1968).

Overall, these and other clinical studies (Section The Clinical Relevance of Hemoglobin, Hemopexin, and Haptoglobin Levels) demonstrate a need to examine the often stated concept, originating from the late 1960's, that HPX is depleted only when Hp is absent (Gladwin and Ofori-Acquah, 2014). HPX levels decline when a substantial amount of heme is present in plasma because as hepatic heme increases, HPX catabolism exceeds *de novo* synthesis in humans and rhesus monkeys (see Section Improving Animal Models of Human Disease in which Hemopexin and Haptoglobin Play a Protective Role). *Thus, to further define the stages and cause of hemolysis and particularly to distinguish impairment of heme clearance from hemolysis, knowledge of plasma heme and HPX levels would enhance the ability of clinicians to define the clinical ramifications*.

## The hemopexin system links heme and iron metabolism

Evidence for this link comes from different experimental approaches in humans and animals. Heme-iron recycling was linked to HPX and heme clearance, by following i.v. ^59^Fe-hematin (0.77–1.23 mg heme/kg body wt.) in IVH from mechanical damage due to aortic and mitral prostheses; IVH from paroxysmal nocturnal hemoglobinemia due to complement destruction of RBCs; and HPX depletion (in the author) after five injections of hematin i.v. (Sears, 1970). Heme bound to HPX and HSA immediately but transfers within 3 min *in vivo* to HPX (Muller-Eberhard et al., 1969; Drabkin, 1971). Body surface counting showed, as expected, most radioactivity in the liver rising over 1–2 days and in the spleen increasing over the first 24 h (~20% dose) before reaching a plateau that was maintained for 21 days. Counts rose over the sacrum within this time supporting rapid re-utilization and ^59^Fe was rapidly detected in the RBCs. There were no changes in plasma HPX, Hp, or bilirubin; and no detected in the urine. In normal subjects, the heme-iron was incorporated into circulating RBCs within 48 h, reached 20% dose in about a days, 37% dose in 3 weeks and 50% dose over 8–12 weeks. This was noted as slightly slower than for Hb-iron re-utilization (Garby and Noyes, 1959) or Hb-iron from effete RBCs (Noyes et al., 1960). Iron status affected heme-iron re-utilization and more than 60% dose after HPX depletion and ~70% in the subject with mechanical hemolysis at 21 days. A detailed analysis was confounded in patients with iron overload due to dilution of the radioisotope by iron stores.

As described previously (Smith, 2011b, 2013), iron status affects the HPX system: the surface receptors for heme-HPX and heme uptake are increased in iron deficiency, and decreased in iron overload (Smith and Ledford, 1988). HPX delivers heme to cells where it is broken down by HOs and the iron released. The heme-iron is stored on ferritin (Davies et al., 1979), or used for metabolic purposes including an iron source for cell growth for which an iron-enzyme ribonucleotide reductase is needed (Smith and Ledford, 1988) or for regulation via the IRP/IRE system of proteins of iron homeostasis in human retinal pigment (Hunt et al., 1996) and human neuroblastoma cells (Hahl et al., 2013).

Oligodendrocytes are the predominant iron-containing cells in the brain, often clustered around a blood vessel, and provide support for CNS neurons. Their only known function is myelin production where iron is a required as a co-factor for cholesterol and lipid biosynthesis, and for oxidative metabolism, which occurs at a higher rate in oligodendrocytes than other types of brain cells (Connor and Menzies, 1996). HPX is proposed to act in the maturation of oligodendrocytes. As HPX^−/−^ mice age, there are fewer mature OLs and more precursor cells although brain iron accumulates. The density of myelinated fibers in the basal ganglia and in the motor and somatosensory cortex of HPX^−/−^ mice decreased at 2 months (equivalent to a young human adult), with decreased neuromotor function (Morello et al., 2011).

## Heme toxicity and hemopexin deficiency states

### Coordination of extracellular and intracellular defenses may be needed under specific circumstances to survive heme toxicity

Oxygen radical reactions occur upon tissue damage and heme with its redox active iron participates in such reactions that can lead to the degradation of proteins, lipids and carbohydrates and nucleotides (DNA and, likely, also RNA). Cells rely on the proteins in extracellular fluids to limit such radical chemistry by converting pro-oxidant forms of iron to less reactive forms (Halliwell and Gutteridge, 1990). HPX is one of these extracellular anti-oxidants as are Hp, transferrin and ceruloplasmin. Only HPX, not Hp, inhibited hemin-stimulated lipid peroxidation providing direct evidence for HPX as the primary extracellular antioxidant protector against heme toxicity in biological fluids (Gutteridge and Smith, 1988). There are numerous examples of this: HPX prevents heme uptake into the aorta (Bui et al., 2004) and endothelial cells while targeting heme to the liver (Smith and Morgan, 1979).

In addition to IVH and EVH, sources of heme *in vivo* would include rupture of atherosclerotic plaques and trivial hemolysis in areas of turbulent blood flow such as the bifurcations of major blood vessels may create areas of sensitized endothelial cells. Heme (1–7 μM) sensitized porcine aortic endothelial cells *in vitro* to subsequent oxidative stress leading to cell death (Balla et al., 1991b). Either a bolus of H_2_O_2_; gradual levels of H_2_O_2_ generated enzymatically by xanthine and xanthine oxidase or ROS produced by phorbol ester activated neutrophils (human polymorphonuclear cells) were toxic. When heme was bound by HPX (i.e., presented as a 1:1 stoichiometric mixture of heme and HPX) there was no endothelial cell toxicity (Balla et al., 1993), as expected because endothelial cells lack of HPX receptors (Smith and Morgan, 1979). Protection against heme toxicity was also conferred by catalase but not by SOD supporting that H_2_O_2_ is the ROS. If cells were incubated with ROS before HO1 and ferritin were induced by heme, the endogenous levels were not effective protection. If ROS exposure occurred a few hours after heme, both proteins confer protection due to their induction by heme and iron, respectively. Several other inducers associated with the inflammatory response and hypoxia are also good inducers of HO1 but not necessarily ferritin protein levels. Some ideal of the temporal relationship is provided by studies albeit in a different cell type. Maximum HO1 protein levels (~2-fold) are induced in mouse hepatoma cells within 3–6 h exposure to heme-HPX, whereas the iron released from heme catabolism may not induce ferritin via translation until ~7–11 h (Alam and Smith, 1989; Sung et al., 2000). Endothelial cell death was accompanied by increased membrane lipid peroxidation. *Endothelial cells are in danger in inflammation with activated neutrophils in close proximity that marginate along endothelial surfaces in presence of diverse inflammatory mediators*.

### Key roles for heme toxicity in the pathogenesis of sickle cell disease and protection by hemopexin

*There has been evidence, some published over 35 years ago, that a lack of plasma HPX, or the presence of heme-HPX with methemalbumin, is an ominous prognosis in sepsis (Larsen et al., 2010; Jung et al., 2015), as well as hemorrhagic shock (Friedman-Mor et al., 1978) and liver cirrhosis (Vladutiu and Kim, 1981)*. SCD is a really important common disease entity and there is experimental evidence from *in vitro* studies with human and rodent cells as well as SCD mice that heme released from metHb is a major cause of several pathological symptoms, which are prevented by HPX *in vitro* and *in vivo*. Plasma heme rises in SCD patients from 0.2 to ~4 μM and may exceed 20 μM (see references in Belcher et al., 2014). Thus, HPX protects by acting as an extracellular anti-oxidant sequestering the heme and targeting the heme-HPX complexes to cells that play a role in iron homeostasis, particularly the liver (Smith and Morgan, 1979). HPX deficiency develops in SCD, in β-thalassemias and in mice models of SCD. Several avenues of how this damage is mediated and how it can be ameliorated have been opened up recently including the modulation by HPX of the inflammatory immune response activated by heme.

In SCD, genetic mutations in globin, generate unstable molecules, HbS, that cause distortions in red blood cell structure, thus increasing their fragility such that shear forces in blood vessel forks and capillary bed cause lysis. The ensuing hemolysis leads to a constant damaging oxidative stress due partly to the chemical reactions that hemoglobin and heme undergo as HPX and Hp levels decline. Painful episodes, or “crises,” are thought to be due to events that precipitate vaso-occlusion in the presence of chronic hemolytic anemia and also an even more rapid hemolysis termed “hyperhemolysis.” Heme drives endothelial cell expression of adhesion molecules to which platelets and RBCs attach, ultimately blocking blood flow. Systemic inflammation occurs simultaneously with ROS release, and, together with ischemia reperfusion injury, which may lead to cerebrovascular compromise (strokes) and organ infarction. The only cure for SCD is bone marrow transplantation, which carries significant risks of morbidity and mortality due to immune suppression or injury induced by the donor immune system recognizing recipient antigens as foreign (graft vs. host disease). Other patient treatments include approaches to avoid vaso-occlusion crises, palliative therapy to manage pain and to relieve symptoms, and antibiotic prophylaxis to prevent infections and pneumonia. New treatments include gene therapy to produce normal Hb, drugs to produce fetal Hb (which lack the abnormal beta subunits that cause hemoglobin polymerization and RBC sickling), NO therapy to prevent sickle cell clumping and statins to limit cholesterol levels and decrease oxidative stress in blood vessels^6^.

HPX deficiency in SCD mice promotes vaso-occlusion (Belcher et al., 2014) and lung injury, i.e., acute chest syndrome (Ghosh et al., 2013). *The extent of any hemolysis is driven by RBC instability and the extent of potential heme toxicity by changes in heme that affect it's binding to globin*. Compared with HbAA mice, the RBCs in HbSS mice turn over rapidly (*t*_1/2_ of 2–5 days) in a steady state of stasis at ~8–10%. Infusions of Hb in the HbSS mice increased stasis to 30%. *Co-administration of either Hp or HPX with Hb prevented stasis, supporting that heme is the active agent*. HbS caused more stasis than HbA consistent with its known instability and supporting that HbS more readily releases heme; thus, enhancing the pathology of SCD. Infusions of HbA into control mice generated low levels of stasis (similar to saline-induced iv hemolysis). Thus, heme induces stasis in sickle, not WT, mice. As platelets and RBCs adhere to the walls of endothelial cells lining blood vessels, the blood flow slows and vaso-occlusion can occur. *Heme must be released from metHbA to cause the pathology* because the stable derivative of heme, cyanometHb, did not cause vaso-occlusion (36.2% stasis vs. 8.2%, respectively); nor did mixtures of HbA with methylene blue (ferro-Hb), or HbA with Hp, or HbA with HPX.

HPX is part of the innate immunity defense and TLRs are part of the innate immune system to restrict pathogen growth. Heme activates TLR4 leading to TNFα production in mice macrophages *in vitro* (Figueiredo et al., 2007). Using TAK-242 to block the TLR4 interaction with its adaptor molecule, and with TLR4^−/−^ cells and mice, TLR4 was shown to be needed for the heme-responsive trafficking of adhesion molecules to the surface of endothelial cells (Belcher et al., 2014). Importantly, this pathway and a role for TLR4 has also been validated in human endothelial cells. HPX prevented the rapid migration of P-selectin and Von Willibrand Factor (VWF) to the cell surface in response to heme. HPX also prevented heme stimulation of Weibel-Palade body degranulation and the increase in surface P-Selectin and VWR after 24 h incubation with either HbS or HbA. This response was related to the amount of heme because the surface expression of P-Selectin and VWF was greater in response to HbS compared with HbA, reflecting the faster rate of heme loss from HbS (Hebbel et al., 1988). Inhibitors of PKC and NADPH-oxidase or inhibition by anti-oxidants such as N-acetyl cysteine and iron chelators provided presumptive evidence for the involvement of ROS and oxidative stress as part of heme-induced WPB degranulation in endothelial cells and consequent stasis. The NF_K_B signaling pathway was also needed in part. Adhesion molecules identified to be involved in heme-induced stasis in the sickle mice are VCAM1, α4β1 integrin, ICAM-1, PECAM-1, E-selectin, and α4β3 integrin. Unexpectedly, in mice, inactivation of one of these adhesion molecules at a time was sufficient to prevent stasis from taking place. Heme stimulation of surface adhesion molecule expression in cultured endothelial cells was recapitulated in NY1DD mice but not controls. P-selectin and VWF expression was detected on venules of the skin, brain, lungs, liver and kidney. The histo-chemistry showing EC activation and vaso-occlusion revealed clear differences in the different tissue capillary beds. Thus, tissue-specific responses of endothelial cells to: heme, heme and ROS, heme and inflammatory cytokine stimuli under either physiological or pathological conditions are expected. Belcher and colleagues (Belcher et al., 2014), pointed out that many local factors influence the different vascular beds e.g., EC heterogeneity, hemolysis, local cytokine milieux, HPX and Hp levels, HO activity, infections and oxygen gradients plus local or global changes in hemolytic rates, infection and hypoxia to trigger vaso-occlusive crisis. To show that *heme-mediated stasis required TLR4 signaling in endothelial cells in vivo, chimeric NY1DD mice were generated that expressed HbS* by transplanting bone marrow from NY1DD mice into irradiated TLR4^−/−^ and TLR4^+/+^ mice. Heme-induced stasis, using heme in the pathological range of 20–60 μM, occurred in the in the NY1DD/β^s^/TLR4^+/+^ mice or non-transplanted NY1DD mice but not in the NY1DD/β^s^/TLR4^−/−^ mice expressing HbS. β^s^ expression is clearly needed for stasis because the heterozygous HbAS mice had levels of stasis that were intermediate between HbAA and HbSS mice.

Although, humans develop more severe lung injury than mice, heme caused lung injury in SCD mice via neutrophil extracellular trap (NET) formation, which was limited by HPX (Chen et al., 2014). NETS are thought to bind and eradicate pathogens but will also trap platelets and RBCs leading to vaso-occlusion. Induction of NET formation in the lungs requires TNFα-activated neutrophils and can precipitate death. These neutrophils have higher levels of intracellular ROS and also generate ROS from myeloperoxidase, whose levels increase in the plasma. Decondensed chromatin is released that has granular enzymes such as elastase bound to it. The plasma of SCD patients has more activated neutrophils than non-sickle plasma, and in crisis contains significantly higher levels of DNA, nucleosome components, and myeloperoxidase. Heme levels were higher in the plasma of TNF-α treated SCD mice compared with controls. Furthermore, heme stimulated TNF-α primed neutrophils to generate NETs and, as with adhesion factor expression, this was related to heme concentration. Significantly, HPX reduced NET release by over 90% when cells were incubated with a mixture of equimolar amounts of heme and HPX. Heme-HSA, but not HbA, caused significant NET production, consistent with their relative affinities for heme. HPX infusion significantly lowered the heme concentration and reduced the number of pulmonary NETs, plasma DNA, nucleosome levels, and decreased MPO activity in SCD mice. Neither heme nor TNF-α alone caused detectable NETs release. *Thus, these heme effects may occur in response to infection, which would in part explain the development of acute chest syndrome in SCD patients suffering from acute infections*.

Based on the known specificity and extremely high affinity of heme binding by HPX, the metHbA-induced stasis in a background of SCD oxidative stress was due to heme and HPX protects. *Thus, in sterile inflammation with heme-TLR4 activation, heme has been proposed as an erythroid Damage-Associated Molecular Pattern molecule* i.e., a DAMP (Gladwin and Ofori-Acquah, 2014).

### Key roles for heme toxicity in the pathogenesis of sepsis and protection by hemopexin

Sepsis is defined as SIRS due to identified or presumed infection and is the leading cause of death in the USA, affecting more than 1 million annually. Of these deaths, 28–50% are estimated to be due to multiple organ dysfunction syndrome^7^. Recovery from sepsis can be difficult and extended, and sepsis survivors may suffer prolonged or life-long disability. People with weakened immune systems, infants, and the elderly are most vulnerable to sepsis, as well as people with chronic illnesses including diabetes, HIV, cancer, and kidney or liver disease. It is reported that 70–90% of Ebola virus victims in West Africa in 2014 succumbed to sepsis with multi-organ failure^8^. This syndrome is the most expensive condition treated in US hospitals and cost >$20.1 billion in 2011^9^. Unfortunately, sepsis remains a clinical diagnosis, due largely to the non-specific signs and symptoms that patients exhibit during the initial phase of illness. Earlier diagnosis and effective treatment of sepsis would save lives. Treatment of sepsis remains limited to supportive care (particularly vasopressive agents and intravenous fluids), rapid initiation of antibiotic therapy, and identification and drainage of sources of infection. *Sepsis-specific treatments have been overall disappointing; however, evidence from mice supports that plasma protein therapies with HPX and /or Hp could be helpful in reducing the severity of illness and mortality*.

Plasma protein therapies are established as plasma volume expanders after burns and in septic shock (HSA) and also infusions of immunoglobulins for certain immune deficiencies. Japanese and German physicians pioneered Hp infusions to prevent renal damage from hemoglobinuria in burns and, in 1977, the severe hemolytic damage accompanying ABO incompatibility (Homann et al., 1977). In CPB surgery (Tanaka et al., 1991), Hp was undetectable after 30–90 min and hemoglobinuria developed with renal tubular injury. When serum free Hb reached >30 mg/dL, Hp administration cleared plasma Hb within 30 min, hemoglobinuria and improved kidney function. Hp infusions together with fluid resuscitation and plasma administration prevented post burn shock and acute renal failure in a young man with severe burn injury with lung damage (Imaizumi et al., 1994). Unfortunately, a second skin graft 51 days later led to death from sepsis with secondary cardiac failure. The Hp infusions used were: 2000 U (2840 mg) for serum free Hb 50 mg/dL (1000 mg/50 kg body wt.) and 4000–8000 U (5680–11,360 mg) for >100 mg Hb/dL (4000 mg Hp/50 kg, Yoshioka et al., 1985; Imaizumi et al., 1994).

**Figure 2:**
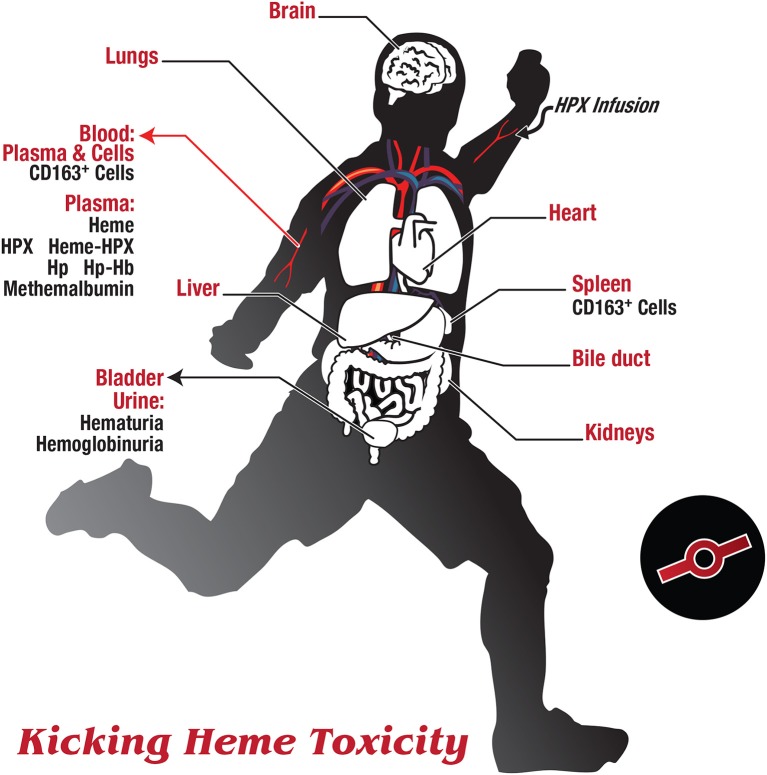
**Kicking heme toxicity**. As originally suggested by Delanghe et al. (2010) and Drieghe et al. (2013), there is at the bedside a currently unrecognized and potentially quite frequent need to distinguish impaired heme clearance from ongoing hemolysis. Several major organs may be involved in the pathology of heme toxicity, including the liver, lungs, heart, spleen, intestine, and kidney. Certain immune-privileged sites such as the brain and gonads are initially protected by strong endothelial barriers with tight junctions, and certain immune-privileged cells exist in an immunosuppressive environment. Thus, endothelial cells provide a physical barrier and epithelial cells provide a selective cell gate. Several markers in blood and urine samples are indicated here, in addition to the standard liver and kidney function tests and parameters used for the hemolytic index. If routinely measured, e.g., free heme, free HPX and free Hp in plasma, and heme in urine should prove useful in diagnosis and help discern when there is impaired heme clearance due to a HPX deficiency state. As endothelial cells die and lyse they may be the principal source of plasma HO1 (Ghosh et al., 2011) and also raise LDH levels Drieghe et al., 2013. The soluble ectodomain (sed) of scavenger receptors are released into the circulation by proteolysis and it is expected that sedCD163 will bind Hb and/or Hp-Hb, and sedLRP1/CD91 may bind heme-HPX.

Two clinical studies provide evidence that low plasma HPX levels at the time of diagnosis are related to the severity of sepsis and indicate a poor prognosis for septic shock. The criteria for sepsis diagnosis used the parameters defined by the American College of Chest Physicians Society of Critical Care Medicine consensus criteria (Moreno et al., 1999). In general, the patients received currently accepted methods of therapy including aggressive fluid resuscitation, broad-spectrum antibiotics during the first 24 h, vasoactive agents; together with one or more doses of hydrocortisone. The mean initial HPX plasma levels in non-survivors (~0.6 mg/ml) were significantly lower than those of the survivors (mean ~1.2 mg/ml; Larsen et al., 2010). In a similar study in Korea, initial serum HPX level in non-survivors was 0.75 mg/ml compared with 1.02 mg/mL (*P* < 0.001) in survivors (Jung et al., 2015). *Thus, based on our current knowledge of how heme toxicity develops, there is every reason to believe that HPX infusions alone, or together with Hp, would be therapeutic in the management of sepsis, septic, and hemorrhagic shock (see Figure 2)*.

*These clinical sepsis data showing that low HPX predicts a poor prognosis provide a rationale for investigating the role of HPX infusions as a therapy for HPX deficiency states secondary to sepsis-induced hemolysis*. The first compelling observations that (i) heme toxicity can be central to the pathogenesis of a human disease allowing heme toxicity to develop as HPX levels decline (i.e., a HPX deficiency state); and (ii) HPX infusions restore circulating HPX levels, reduced mortality, and thus may be a novel therapeutic approach came from studies using mice models of sepsis (Larsen et al., 2010). The foundation was research with Hmox^−/−^ mice, which revealed that HO-1 induction and heme catabolism in response to microbial infection suppressed the development of severe sepsis. Protection was found to be unrelated to the extent of the pathogen load but was related to protection against free heme released by hemolysis during the infection. Thus, non-protein bound heme compromised the host tolerance to infection.

Protection against heme toxicity by HPX also requires HOs and their enzyme activity because protection against heme and heme plus ROS is lost in HO-1^−/−^ neurons and when HO-1 activity is inhibited by tin-protoporphyrinIX (Li et al., 2009). Although protection against redox-active iron was not part of the studies, earlier work (see Section Coordination of Extracellular and Intracellular Defenses May Be Needed under Specific Circumstances to Survive Heme Toxicity) and extended more recently (Vercellotti et al., 2014) showed a key role for the ferroxidase H-ferritin subunit in spleen and liver to protect cells in SCD mice; but considered independent of HO-1 activity (Vercellotti et al., 2014).

Necrosis after toxic heme levels requires several hours so cells initially appear normal and any responses to heme in the short term need to be distinguished from possible toxic effects that only become apparent much later (e.g., after 24 h). HPX also protects cells from “heme sensitization,” the apoptotic cytotoxic effect of heme that requires exposure of cells not only to heme but subsequently to pro-inflammatory molecules such as TNF. Uptake of heme-HPX by primary human or mouse hepatocytes did not raise ROS levels as did heme, even after subsequent incubation with TNF for 16 h (Larsen et al., 2010). TNF alone raised release of HMGB1 from these primary hepatocytes and this was exacerbated by previous incubation with heme but not with heme bound to HPX. *Thus, HPX prevents direct cytotoxicity of heme and heme sensitization of cells that is one consequence of heme exposure*.

Low-grade sepsis in response to colon puncture and ligation is toxic in Hmox1^−/−^ mice but not WT mice (Hmox1^+/+^) and i.v. heme promotes tissues damage and leads to a severe “high grade” sepsis (Larsen et al., 2010). This takes place although the levels of cytokines produced in the septic inflammatory response e.g., TNF, IL-6, and IL-10, were similar in Hmox1^−/−^ and WT mice. Hb levels were above normal in Hmox^−/−^ but not the Hmox^−/+^mice. Rodent models of human diseases where HPX and Hp are protective are complicated by the fact sham operations, stress, and inflammation alone can lead to significant increases in both HPX and Hp plasma levels. Nevertheless, the HPX levels were highest in the WT mice, decreased by about 20% in Hmox1^+/−^ (from ~2.45 to 2 mg/ml) and had further decreased but were still detectable in the Hmox1^−/−^ mice Mice start to die as early as 2 days after CLP; however, HPX infusions (50 mg/kg ip) caused a significant decrease in this morbidity from 75 to 20% when given at 2, 12, 24, and 36 h post CLP. The control mice were given IgG (50 mg/kg ip) and an equal volume of the solvent control, PBS.

### Heme toxicity in hemorrhagic shock and links with hemopexin

Although hemorrhagic shock differs from septic shock (Wichterman et al., 1980), HPX metabolism is altered in hemorrhagic fever from Dengue virus, which is transmitted by infected mosquitoes. HPX levels were 10% of controls in severe dengue associated with hemorrhagic fever (DHF) and a rapid drop in platelets, during a dengue epidemic in Thailand (Muller-Eberhard and Liem, 1975). HPX levels may help determine the severity of the illness, discriminate between dengue hemorrhagic fever (DHF) and uncomplicated dengue fever (DF), which would aid in diagnosis and treatment; and fibronectin, HPX and transferrin were found to be significantly decreased in DHF (Kumar et al., 2012). In other studies, HPX and vitronectin were increased in DF and DHF (Poole-Smith et al., 2014). Although by no means proven [see references in (Poole-Smith et al., 2014) on data from other human and swine studies], *HPX together with other biomarkers such as Hp, may prove useful as a supplement in certain stages of hemorrhagic shock and even, potentially in the progression of Ebola in patients, especially those that progress to hemorrhage*.

## Improving animal models of human disease in which hemopexin and haptoglobin play a protective role

Experimental animals allow investigation of tissues not readily available in humans as well as greater control over response, sampling, and treatment times; thus, standardization is of prime importance in good animal models of human disease. The rhesus monkey (rhesus macaque, *Macaca mulatta*) is a primate model with the most similar genetics, physiology, and metabolism to humans. In contrast, with respect to the response to infection, mice can tolerate far larger burdens of bacteria than humans without succumbing and although this differential response can be bacterial species specific, there are differences between humans and mice in their response to inflammatory conditions (Seok et al., 2013). In mice and rats, HPX acts as an acute phase reactant and there are very rapid and extensive increases in plasma levels of HPX and Hp (e.g., ~4–5-fold in inflammation) induced by a variety of stimuli including sham surgery. These are far higher than the ~1.5–2-fold changes documented in humans in various diseases and conditions. In general, animal plasma levels of HPX (mg/100 ml) are similar to humans (Drabkin, 1971; Lane et al., 1973), e.g., rabbit (31–52), rat (63–82), and dog (76–128). *However, in humans, HPX is not an acute phase reactant and was not induced by muscle biopsy that caused an inflammatory response raising Hp among other standard parameters e.g., fibrinogen and α_1_-antitrypsin (Elin et al., 1982)*.

Human HPX is a highly conserved glycoprotein and uniquely has an N-terminal O-linked oligosaccharide in addition to five bi-antennary chains (for review, see Smith, 2011b). Heme coordination is identical in human, rabbit, bovine, and rat HPX based on a variety of physic-chemical data including absorbance, electron paramagnetic resonance and magnetic circular dichroism spectroscopy. However, there are clear differences between the HPX and Hp systems in humans and in rats including species differences in receptor affinity.

One way forward to better understand the metabolism and the roles of HPX and Hp in humans is to compare directly the structure and function of human and mouse proteins and their receptor interactions *in vivo* and in cultured cells *in vitro*. Significant differences in the high affinity of human Hp-Hb for human HuCD163 compared with that of mouse Hp-Hb for mouse CD163; and between the high affinity of mouse Hb for mouse CD163 compared with human Hb for human CD163 as well as *in vivo* uptake supported that in humans Hb clearance is principally via receptor-mediated uptake of Hp-Hb but not in mice (Etzerodt et al., 2013). *It would seem that additional studies in humans are still needed to determine the extent of involvement and relative contributions of the role of the liver and macrophages in Hp-Hb clearance and at what times during the course of the development of disease-related pathology*.

The normal plasma clearance in rats of human and rabbit HPX is faster than that of rat HPX (Liem, 1976). There are differences in the affinity of human, rabbit and rat HPX for rat hepatic HPX receptors that affects the rate of heme clearance from the circulation and the extent of liver heme uptake from ^59^Fe-heme bound to ^125^I-labeled human, rabbit or rat HPX in rats (Smith and Morgan, 1979). More complex was taken up and more heme delivered to the liver within 5–15 min in the order human > rabbit > rat HPX complexes supporting that human HPX has a higher affinity for the rat HPX receptors than rat HPX. All three species of HPX interact with a common receptor on liver cells because increasing the levels of circulating complexes, led to similar hepatic saturation levels [~200 pmol/g of liver for both rat and rabbit HPX (Smith and Morgan, 1978)]; and competitive inhibition studies showed that non-radiolabeled rabbit heme-HPX complexes blocked radiolabeled rat heme-hemopexin uptake. In these experiments, no extensive catabolism of ^125^I-rabbit HPX occurred; supporting that the chloramine-T iodination, carried out under oxidizing conditions, had not denatured the protein which would then have been rapidly degraded by the liver. Furthermore, the heme levels used were low enough to minimally affect HPX metabolism.

Endothelial cells are clearly a heme target in SCD; also HO1 is increased in renal tissues of SCD patients but not in the Townes and Berkeley mice model of SCD (that are on identical backgrounds, Ghosh et al., 2011). Circulating endothelial cells, shed from the vascular wall lining into the bloodstream, are rarely seen in normal individuals but are in SCD and may reflect vascular damage/dysfunction. The responses of cytoprotective enzymes to heme *in vitro* were found to vary depending upon the duration of heme exposure and origin of endothelial cells. Primary human endothelial cells from the pulmonary artery (PAECs) and microvasculature (PMVECs), and dermal microvasculature were incubated with multiple exposures to heme (5–25 μM), considered not toxic, in fresh media every 48 h for 7 days. Five genes differentially regulated by heme in PMVECS and PAECs were shared: HO1, glutamate-cysteine-ligase modifier subunit, NQO1, H-ferritin and δ-aminolevulinate synthase 1. Nrf2 regulation was considered central: whereas HO1 was quickly induced by heme, NQO1 required several days and was apparent only in the older mice perhaps due to the oxidant burden of the disease and low levels of endothelial SOD. NQO1 staining was more intense in clinical SCD than in mice. One similarity between the mice and clinical SCD was the intensity of HO1 staining in post-mortem lung endothelium, although HO1 mRNA levels were highly variable in the patients. Plasma HO1 levels from SCD patients varied considerably, but correlated with markers of endothelial cell activation and injury. These enzyme changes may provide a strategy for additional SCD therapy (Ghosh et al., 2011).

Patients and rhesus monkeys were used to investigate why serum levels of HPX are abnormal in hemolytic anemias, chronic neuromuscular diseases, and acute intermittent porphyria. The synthetic and fractional catabolic rates of HPX were measured in patients exhibiting low, normal, or elevated serum HPX levels (Foidart et al., 1983). Elevated HPX levels principally reflected increased synthesis not decreased catabolism of HPX: the mean synthetic rate (13 ± 1.0 mg/kg/day) was twice that of the patients with normal HPX levels (6.6 ± 0.3 mg/kg/day), whereas the fractional catabolic rate was ~ 33% higher than controls (i.e., 35.3 ± 7.1% of the i.v. pool per day compared with 26.5 ± 0.8 for controls). Low serum HPX in patients with SCD appeared to be due to increased HPX catabolism (36.0 and 40.0% of the i.v. pool per day vs. 26.5 ± 0.8 for controls) without compensatory synthesis. Clinical conditions in which serum HPX levels are either increased (acute intermittent porphyria, chronic neuromuscular diseases) or decreased (severe hemolysis or rhabdomyolysis) were simulated in rhesus monkeys by administering heme i.v. daily over 10 days (Foidart et al., 1982). Low heme (0.02–0.04 mg/kg/day) increased serum HPX levels by 50% (control = 53.3 ± 2.8 U/100 ml) due to a 76% increase in the net rate of HPX synthesis, that enlarged the IVH pool size of HPX by 65%. An intermediate heme dose (0.6 mg/kg/day) caused a 43% increase in the rate of HPX synthesis balanced by a 33% increase in catabolism; and HPX serum levels were unchanged. High heme (5.0 mg/kg/day) decreased HPX to 60% of control due to a 57% increase in HPX catabolism without a concurrent increase in synthesis. HPX metabolism reached steady state within 4 days of heme exposure; and both ^125^I-human and monkey HPX were metabolized in the same way. HPX was specifically regulated by heme because plasma transferrin and Hp were unaltered. *These findings support a relationship between the amount of heme presented to the liver and net HPX turnover; and implicated, for the first time, a role for hepatic heme levels in HPX regulation*. Consistent with these observations, heme induces HPX mRNA in murine hepatoma cells (Smith, 1999) and primary embryonic rat neurons (He et al., 2010). From other studies but relevant here, raising plasma heme did not alter the half-life of HSA as it does HPX. Furthermore, HSA was cleared faster than rabbit albumin with half-lives of ^125^I-HSA and heme-^125^I-HSA compared with rabbit albumin and heme-rabbit albumin [3.25–3.75 days compared with 4.5–5.25 days (Liem et al., 1975)].

This research in monkeys demonstrates the advantage of investigating the response of HPX to a wide range of heme concentrations based upon human studies, especially low levels. Most studies use a single, pharmacologically toxic high heme dose, e.g., a 50-fold excess of theoretical/possible HPX binding (Lane et al., 1973). Heme is a normal constituent of bile in humans 12.6 nmol/h compared with 8.2 μmol/h bilirubin. Heme administration (6.4 μmol/kg) increases heme (109.5 nmol/h) not bilirubin (McCormack et al., 1982). Higher amounts of heme administered i.p. in rats (5–40 mg/kg body weight) causes bile to turn black [heme at 0.29 mg/ml (Petryka et al., 1977)]. However, whether this is due to unregulated diffusion via the FLVCR heme exporter or a normal entero-hepatic circulation of heme is not established. In rats, at low doses of heme (47 nmol/100 g body weight) based upon 10 × the daily heme requirement of humans relative to body weight, there is extensive heme catabolism in duodenal enterocytes. *Because only iron reached the circulation bound to transferrin, no heme-HPX was detectable, these data revealed that duodenal enterocytes are the first point of convergence of heme and iron metabolism in the body (Smith, 1990, 2009, 2011b).*

The genetic background of mice affects their iron status, and the iron status of mice and their strain affects Hp expression in different ways depending upon the response of the ER unfolded protein pathway to iron load (Faye et al., 2007). There was a significant decrease in liver and serum Hp in C57BL6 mice but not the 129sv strain, fed an iron-rich diet. The chaperone for protein folding in the ER, glucose regulated protein, GRP78, was decreased in the C57BL6, but not in the 129sv, mice. Iron overload negatively impacted Hp synthesis likely by creating oxidative stress in the ER. Then incorrectly processed and folded Hp is degraded upon GRP78 depletion. It is not known if this affects the metabolism of Hp, whose regulation is stabilized in the HPX^−/−^ mice (Tolosano et al., 1999), which are on the 129sv background.

*Overall, these animal studies demonstrate and re-inforce the conservation across species of the protective role of HPX against heme toxicity, the development of HPX deficiency states, and show how raising heme and iron levels in the liver makes an impact on HPX and Hp synthesis and metabolism*.

## Concluding remarks

In this article we have outlined the deleterious physiological consequences of IVH free heme and the role these effects play in the development and complications of various disease processes in humans. HPX's actions, biological roles, metabolism, and function have also been studied in rhesus monkeys, dogs, rabbits, rats, and HPX^−/−^ mice (Tolosano et al., 1999, 2002; Morello et al., 2007; Fagoonee et al., 2008; Vinchi et al., 2008; Li et al., 2009; Chen et al., 2011).

Relevant to human disease: (1) HPX sequesters heme from cells preventing direct cytotoxicity and heme sensitization; and in cells lacking HPX receptors this prevents endothelial and immune cell activation leading to stasis and exacerbation of the inflammatory response, respectively; (2) Plasma HPX targets heme principally to the liver and normally recycles intact after receptor-mediated endocytosis while the heme is degraded and its iron re-claimed; (3) Hepatic uptake of heme-HPX is via a high affinity low capacity system, likely LRP1/CD91, and a low affinity high capacity system (Smith and Morgan, 1981; Smith, 2013); (4) Heme transfers rapidly in plasma from albumin to HPX; (5) Plasma HPX levels are linked to plasma and hepatic heme, not plasma Hb levels or the acute phase response; (6) Changes in plasma HPX levels reflect the balance of hepatic synthesis and secretion of HPX with HPX catabolism; HPX declines with increased hepatic heme as HPX catabolism overshadows compensatory synthesis; (8) HPX metabolism responds to heme from Hb and Mb; (9) HPX protects erythrophagocytosing macrophages from heme toxicity in EVH; (10) HPX functions independently of and simultaneously with Hp and does not require global Hp depletion. As we strive to improve animal models, two current challenges to accurately describing Hb metabolism lie in the differences in Hb-Hp metabolism in CD163^+^ human macrophages compared with these cells in mice and the lack of a reliable, high throughput assay to detect and quantitate in plasma Hb-Hp, heme-HPX, and methemalbumin.

Incorporating this contemporary understanding of heme metabolism into the evaluation of disease states prominently characterized by hemolysis may likely prove valuable in a variety of ways, including: (1) providing accurate diagnosis of acute illness; (2) improving our ability to determine which patients are at greater risk for more severe illness, complications, or death; and (3) developing targeted replenishment therapies for patients suffering from acute illnesses due to, or exacerbated by, significant hemolysis. However, large gaps remain in our understanding of heme metabolism and the role that HPX and Hp could play in specific disease states. In addition to the bench-side experiments using animal models that have provided much of the knowledge to date on the roles of HPX and Hp in heme and Hb metabolism, respectively, translational approaches that use human-derived samples from patients with and without specific diseases and/or risk factors are warranted.

Future studies in human subjects should focus on several common diseases that often feature, or are complicated by, hemolysis. These diseases include SCD, sepsis, hemolytic anemias, and viral hemorrhagic fevers (e.g., Dengue, Ebola, and Marburg). Sepsis and SCD in particular have readily-available animal models and baseline data, and disease incidence is sufficiently common to allow both acute and longitudinal studies in humans. Future studies should be conducted to determine HPX levels in these disease states, to assess the discriminatory ability of HPX for disease in symptomatic and/or at-risk individuals, and to determine the prognostic utility of obtaining HPX levels in acutely-ill patients. When assessing severe illness characterized by prominent hemolysis, physicians currently use hematological profiles that quantitate cellular components and measure coagulation function. We propose that including plasma heme and HPX measurements as adjuncts to these standard hematological assays may further enhance the diagnostic and prognostic capabilities of the hematological profile, thereby enhancing clinical decision-making. An international standard for plasma heme and HPX, as previously suggested (Delanghe and Langlois, 2001), is called for and will make defining heme toxicity risk more accessible and easier to interpret. Finally, many disease states characterized prominently by hemolysis lack targeted therapies. Investigating the role of replenishment therapies, particularly with HPX to sequester heme, may prove beneficial for these patients and in a plethora of clinical situations including cardiac surgery, blood transfusions, and patients with a high risk of developing sepsis (e.g., with burns, trauma injury, bed sores in the intensive care units or who have alcoholism).

## Author contributions

Both authors wrote and edited the manuscript; AS designed the figures.

### Conflict of interest statement

The authors declare that the research was conducted in the absence of any commercial or financial relationships that could be construed as a potential conflict of interest.

## Footnotes

^1^FDA Workshop on artificial hemoglobin. http://www.fda.gov/downloads/BiologicsBloodVaccines/NewsEvents/WorkshopsMeetingsConferences/TranscriptsMinutes/UCM051545.pdf.

^2^Workshop on artificial hemoglobin. http://www.fda.gov/downloads/BiologicsBloodVaccines/NewsEvents/WorkshopsMeetingsConferences/TranscriptsMinutes/UCM051545.pdf.

^3^http://www.uptodate.com/contents/approach-to-the-diagnosis-of-hemolytic-anemia-in-the-adult

^4^http://www.redcrossblood.org/learn-about-blood/blood-facts-and-statistics#blood-components$

^5^http://anthro.palomar.edu/blood/blood_components.htm

^6^http://www.mayoclinic.org/diseases-conditions/sickle-cell-anemia/basics/treatment/con-20019348.

^7^http://www.nigms.nih.gov/Education/Pages/factsheet_sepsis.aspx

^8^http://world-sepsis-day.org/?MET=SHOWCONTAINER&vCONTAINERID=1032.

^9^http://www.infectioncontroltoday.com/news/2014/04/cdc-offers-new-sepsis-fact-sheet.aspx.

## Abbreviations

Mr: Apparent Molecular weight
CPB: Cardiopulmonary bypass
CD91/LRP1: CD163, Cluster of differentiation
DF: Dengue fever
DHF: Dengue hemorrhagic fever
EVH: Extravascular hemolysis
FLVCR: Feline leukemia virus Sub group C receptor
FPN: Ferroportin
Hp: Haptoglobin
HO: Heme oxygenase
HPX: Hemopexin
HIV: Human immunodeficiency virus
IVH: Intravascular hemolysis
IL-6: interleukin-6
MetHb: Methemoglobin
Mb: Myoglobin
NQO1: NADP(H):quinone oxido-reductase 1
NET: Neutrophil extracellular trap
NO: Nitric oxide
NOS: Nitric oxide synthase
PMVEC: Pulmonary artery endothelial cell
PMVEC: Pulmonary microvasculature endothelial cell
PHP: pyridoxalated Hb polyoxethylene conjugate
ROS: Reactive oxygen species
RBC: red blood cell
SCD: Sickle cell disease
TNF: tumor necrosis factor
VWF: Von Willibrand Factor.
